# Cemiplimab in an Elderly Frail Population of Patients With Locally Advanced or Metastatic Cutaneous Squamous Cell Carcinoma: A Single-Center Real-Life Experience From Italy

**DOI:** 10.3389/fonc.2021.686308

**Published:** 2021-11-08

**Authors:** Sabino Strippoli, Annarita Fanizzi, Davide Quaresmini, Annalisa Nardone, Andrea Armenio, Francesco Figliuolo, Raffaele Filotico, Livia Fucci, Fabio Mele, Michele Traversa, Federica De Luca, Elisabetta Sara Montagna, Eustachio Ruggieri, Simona Ferraiuolo, Francesco Macina, Stefania Tommasi, Angela Monica Sciacovelli, Ivana De Risi, Anna Albano, Raffaella Massafra, Michele Guida

**Affiliations:** ^1^ Rare Tumors and Melanoma Unit, Istituto di Ricovero e Cura a Carattere Scientifico (IRCCS) Istituto Tumori Giovanni Paolo II, Bari, Italy; ^2^ Health Physics Unit, Istituto di Ricovero e Cura a Carattere Scientifico (IRCCS) Istituto Tumori Giovanni Paolo II, Bari, Italy; ^3^ Radiotherapy Unit, Istituto di Ricovero e Cura a Carattere Scientifico (IRCCS) Istituto Tumori Giovanni Paolo II, Bari, Italy; ^4^ Plastic Surgery Unit, Istituto di Ricovero e Cura a Carattere Scientifico (IRCCS) Istituto Tumori Giovanni Paolo II, Bari, Italy; ^5^ Dermatology Unit, Istituto di Ricovero e Cura a Carattere Scientifico (IRCCS) Istituto Tumori Giovanni Paolo II, Bari, Italy; ^6^ Pathology Unit, Istituto di Ricovero e Cura a Carattere Scientifico (IRCCS) Istituto Tumori Giovanni Paolo II, Bari, Italy; ^7^ Radiology Unit, Istituto di Ricovero e Cura a Carattere Scientifico (IRCCS) Istituto Tumori Giovanni Paolo II, Bari, Italy; ^8^ Medical Oncology Unit “Don Tonino Bello”, Istituto di Ricovero e Cura a Carattere Scientifico (IRCCS) Istituto Tumori Giovanni Paolo II, Bari, Italy; ^9^ General Surgery Unit, Istituto di Ricovero e Cura a Carattere Scientifico (IRCCS) Istituto Tumori Giovanni Paolo II, Bari, Italy; ^10^ Pharmacy Unit, Istituto di Ricovero e Cura a Carattere Scientifico (IRCCS) Istituto Tumori Giovanni Paolo II, Bari, Italy; ^11^ Interventional and Medical Oncology Unit, Istituto di Ricovero e Cura a Carattere Scientifico (IRCCS) Istituto Tumori Giovanni Paolo II, Bari, Italy; ^12^ Pharmacogenetics and Molecular Diagnostic Unit, Istituto di Ricovero e Cura a Carattere Scientifico (IRCCS) Istituto Tumori Giovanni Paolo II, Bari, Italy

**Keywords:** cemiplimab, advanced cutaneous squamous cell carcinoma, checkpoint inhibitors, elderly patients, immunocompromised patients

## Abstract

**Background:**

Cutaneous squamous cell carcinoma (CSCC) is the second most common skin cancer whose incidence is growing parallel to the lengthening of the average lifespan. Cemiplimab, an antiPD-1 monoclonal antibody, is the first approved immunotherapy for patients with locally advanced CSCC (laCSCC) or metastatic CSCC (mCSCC) thanks to phase I and II studies showing high antitumor activity and good tolerability. Nevertheless, at present, very few data are available regarding cemiplimab in real-life experience and in frail, elderly, and immunosuppressed patients as well as regarding biomarkers able to predict response so as to guide therapeutic choices.

**Patients and Methods:**

We built a retroprospective cohort study including 30 non-selected patients with laCSCC (25) and mCSCC (five) treated with cemiplimab from August 2019 to November 2020. Clinical outcomes, toxicity profile, and correlations with disease, patients, and peripheral blood parameters are explored.

**Results:**

The median age was 81 years (range, 36–95), with 24 males and five patients having an immunosuppressive condition, while the frailty prevalence was 83% based on index derived from age, Eastern Cooperative Oncology Group performance status, and Charlson Comorbidity Index. We reported 23 responses (76.7%) with nine complete responses (30%). A statistically significant higher response rate was observed in head and neck primary tumors and in patients with hemoglobin level >12 g/dl. No difference was observed with respect to frailty, median age, sex, and body mass index. The baseline low neuthophil/lymphocyte ratio and low platelet/lymphocyte ratio resulted to be also correlated with a better response. Moreover, lymphocyte, neutrophil, and monocyte behaviors had an opposite trend in responders and non-responders. An overall response was reported in four of five immunosuppressed patients. Seventeen patients (57.6%) have an ongoing response and are still alive. Six responders had interrupted treatment (two for toxicity and four for personal choice) but maintained their response. The treatment was well tolerated by the majority of patients. The most common adverse events were fatigue in seven patients (23.3%) and skin toxicity in 10 patients (33.3%), including pruritus in six patients, rash in three patients, and bullous erythema in one patient.

**Conclusions:**

In our real-life experience, cemiplimab showed a high antitumor activity with acceptable safety profile similar to those in trials with selected patients. Moreover, its antitumor activity resulted to be not impaired in very elderly patients and in those with immunocompromised status.

## Introduction

After basal cell carcinoma, among non-melanoma skin cancers, the second most common cancer is cutaneous squamous cell carcinoma (CSCC), whose incidence rates are dramatically increasing over the last decades (1–4).

The risk of developing CSCC increases with age and could depend on the lifetime accumulation of ultraviolet (UV) radiation damage and the onset of immune suppression such as in patients with immunodeficiency virus infection, hematological neoplasms, or autoimmune diseases treated with immunosuppressive agents (5, 6).

The standard therapy for localized CSCC is surgery eventually associated to complementary radiotherapy, but in some cases this approach is not sufficient or not feasible due to locally advanced extent at onset (7). In addition, approximately 5% of patients are metastatic at diagnosis or develop metastases or inoperable local recurrence after complete excision. For these patients, until recently, the standard treatment was platinum-based chemotherapy, but it provided disappointing results and short duration of responses. Moreover, the heavily toxic profile of these drugs compromised the quality of life of patients, often elderly and with several significant comorbidities, requiring dose reductions or a definitive suspension of treatments (8–10). The epidermal growth factor receptor inhibitor cetuximab was also used as first-line single drug, but it showed limited efficacy in advanced CSCC (11).

Of note is that it has been demonstrated that CSCC is characterized by a high mutational load capable of inducing a high expression of tumor neoantigens, making this tumor suitable for immunotherapy (12–14). This therapy meets its biological rational also in the accumulation of immune inhibitory molecules such as programmed death-1 (PD-1) ligand in the microenvironment during tumor growth (15, 16).

Recently, cemiplimab, a monoclonal antibody against the PD-1 receptor, has been approved in the US and EU for patients with locally advanced CSCC (laCSCC) or metastatic CSCC (mCSCC) unfit for curative surgery or radiotherapy. In fact, phases I and II studies reported a significant antitumor activity of cemiplimab in about half of patients regardless of PDL1 expression or extent of total genetic mutation burden, with an acceptable toxicity profile (17). However, these studies recruited selected patients, with the exclusion of those with immunosuppressive status such as transplant recipients and patients with hematological diseases or relevant comorbidities and organ function alterations, as often seen in the elderly population. These clinical features are often found in the real-world population of advanced CSCC and collectively define frailty as a common vulnerability condition among older cancer patients which is associated to an increased risk for poor therapeutic outcomes (18, 19). Thus, due to the limited data still available in non-selected patients (14, 20–24), in this paper, we report our experience with cemiplimab in a frail population treated outside controlled clinical trials and including very elderly patients presenting with several co-morbidities and patients with immunosuppressive conditions requiring a careful assessment of the cost–benefit profile of treatment. Moreover, owing to the absence of biomarkers able to predict response that would guide the therapeutic choice, we investigated the correlations between therapy outcomes and both clinical and blood parameters. The role of simple blood parameters was previously explored in a cemiplimab series (22) showing a predictive value of the absolute lymphocyte count and was established in various cancer settings (25) in which specific white cells and their ratios, like neutrophil-to-lymphocyte ratio (NLR) and platelet-to-lymphocyte ratio (PLR), were shown to mirror the complex interplay between thrombosis, inflammation, and immunosuppression (26, 27). Thus, we assessed the predictive role of blood count both as pre-treatment value and as longitudinal variations.

## Patients and Method

### Patients and Study Design

We built an observational cohort study by retrospectively reviewing the medical records of 30 consecutive patients with laCSCC or mCSCC treated at the “Giovanni Paolo II” National Cancer Institute of Bari, Italy, from August 2019 to January 2021. Among these patients, 19 began treatment within a compassionate use program made available by Sanofi-Regeneron Company until the official approval by the Italian Regulatory Agency in May 2020. Cemiplimab was administered at a flat dose of 350 mg every 21 days until disease progression or unacceptable toxicity.

The screened patients were 18 years or older with histologically confirmed laCSCC or mCSCC. The patients were evaluated if their medical records reported the Eastern Cooperative Oncology Group (ECOG) performance status (PS), a complete medical and therapeutic anamnesis, and at least one measurable target lesion, including visible CSCC lesions as documented by digital medical photography or any other evaluable lesion at radiological imaging according to Response Evaluation Criteria in Solid Tumors, version 1.1 (RECIST 1.1) (28).

Clinical evaluation from the multidisciplinary tumor board was required to confirm that the patients were unfit for surgery or radiotherapy. During the cemiplimab therapy, the addition of local treatment was allowed according to a subsequent board evaluation due to shrinkage, making the lesions suitable of these therapies or due to palliative intents. These patients were included in the analysis if they achieved a RECIST response to cemiplimab before the addition of local treatment. Disease staging was performed prior to treatment and included a total body CT scan for all patients. All patients underwent baseline laboratory tests to assess the main organ functions, including a complete blood cell count and a complete metabolic panel with serum creatinine, blood urea nitrogen, aspartate aminotransferase, alanine aminotransferase, and total bilirubin. The same tests were performed during treatment as standard laboratory care. Moreover, the TSH, fT3, fT4, ACTH, and cortisol levels were regularly sampled to detect any possible immune-related adverse event early. In our study, all patients treated with cemiplimab were included irrespective of the presence of alterations in bone marrow, renal, liver, cardiac, pulmonary, and endocrinological function. Chronic liver viral infections were allowed, provided that the patients were strictly monitored.

The patients were not included in the analysis if they were treated prior with anti-PD-1 or anti-PD-L1 therapy and had active concurrent malignancies other than cutaneous squamous cell carcinoma. However, enrollment was allowed for patients with stable hematological malignancies, adequately treated basal cell carcinoma of the skin, *in situ* carcinoma of the cervix, *in situ* ductal carcinoma of the breast, and low-risk early-stage prostate adenocarcinoma under active surveillance. All the patients included in the analysis signed a written informed consent as part of the study as previously approved by the ethics committee of the IRCCS Istituto Tumouri Giovanni Paolo II, Bari, Italy (Prot. 590/16 C.E.). Sixteen of our 30 patients were also included in an Italian multicenter study (24).

### Procedures

The patients received cemiplimab intravenously over 30 min at a flat dose of 350 mg every 21 days until disease progression, unacceptable toxicity, withdrawal of consent, or at the discretion of the physician if continuing the treatment could put the patient at risk or if it was deemed in the best interest of the patient, considering a balance between the benefits and the risks of treatment.

The assessments of tumor response were performed every two cycles by photographs of the superficial lesions and every 3 months by CT or MRI scan of laCSCC, while for mCSCC tumor assessment this was performed every 3 months by radiological evaluation. All responses were confirmed at least 4 weeks after the criteria for response were initially met: all responses that were not confirmed at the following evaluation were considered stable diseases at the assessment of best overall response. Treatment beyond progression was allowed in case of clinical benefit at the discretion of the clinician.

All patients who received at least one dose of cemiplimab were assessed for safety. Toxicity assessments included reporting of laboratory monitoring, clinical parameters, and treatment-related adverse events graded according to the National Cancer Institute Common Terminology Criteria for Adverse Events, version 5.0 (CTCAE v5.0). Treatment interruptions were allowed in case of grade 3 or higher treatment-related adverse events. The patients were considered for resumption of treatment once the treatment-related adverse event resolved to grade 1 or baseline. Otherwise, treatment was discontinued, and the patient was addressed to regular clinical and radiologic follow-up.

Standard peripheral blood parameters (total leukocyte count, neutrophils, lymphocytes, monocytes, hemoglobin, platelets, NLR, and PLR) were registered before treatment and after 1, 2, 3, and 6 months from the start of immunotherapy to verify correlations between these hematological features and clinical outcomes.

Medical data were reviewed to categorize the comorbidity of a patient by the modified Charlson Comorbidity Index (CCI) (29). A frailty index adopted in studies on a similar population (30–32) was set up by adding scores assigned to age, ECOG performance status, and CCI as defined in **Supplementary Table S1**. A score ≥2 defined the frail population.

### Outcomes

We firstly assessed the best overall response, considering the proportion of patients with complete or partial response (overall response rate, ORR) and the proportion of non-progressing patients (disease control rate, DCR). We then evaluated the time between the start of treatment and the first date of recurrent or progressive disease or death from any cause (progression-free survival, PFS) and the time between the onset of complete or partial response and the first evidence of recurrent or progressive disease or death for any cause (duration of response, DOR). We also assessed overall the survival (OS); safety and tolerability were registered and graded as well according to the CTCAE 5.0 classification of adverse events. Finally, we performed a statistical analysis to assess the possible correlations between therapy outcomes and disease and patient characteristics and hematological parameters.

### Data Collection

Clinical data from medical records were collected in an anonymized database including the characteristics of patients (sex, age at diagnosis and at metastatic disease, significant comorbidities, previous treatments, and PS), the features of the disease (primary tumor site, grade of differentiation, tumor size, disease free interval, and stage), clinical outcomes (response and duration, PFS, and OS), and adverse events. Peripheral blood tests were also collected and analyzed.

### Statistical Analyses

The results are presented according to the intention-to-treat principle. The proportion of patients achieving an objective response, stable disease, or progressive disease was evaluated according to clinical or RECIST 1.1 criteria and analyzed in descriptive statistics.

The duration of response, PFS, and OS were estimated by the Kaplan–Meier method. For DOR, patients with complete or partial response without disease progression were censored at the time of their last valid tumor assessment. Similarly, patients without disease recurrence or progression and patients alive at their last tumor assessment were censored from PFS and OS, respectively.

The association between ORR and age, hemoglobin, total leukocytes, neutrophils, lymphocytes, monocytes, platelets, NLR, and PLR was measured on an interval scale and analyzed with the Mann–Whitney non-parametric test, whereas all other features in the ordinal and nominal scale were analyzed with the chi-square test.

The variations of blood count parameters were registered and compared between the responder and the non-responder subcohorts. Furthermore, basal NLR and PLR were dichotomized according to their median values as low or high; then, the combinations of their values were correlated with response. Results were considered statistically significant for *p*-values inferior to 0.05.

## Results

### Patients’ Population

The main baseline characteristics of the study population are summarized in **Table 1**. Briefly, the main features of our cohort were male sex (80%) and median age of 81 years (range, 36–95), with a prevalence of frailty of 83% according to the adopted index and a median CCI of 2 (range, 0–5). Mostly, there were laCSCC (83.3%) located at the head and neck region (23 patients, 76.7%) that had undergone at least one surgery for CSCC. Only 33% of patients had been previously treated with radiotherapy, while 3 patients underwent subsequent concomitant radiotherapy after completing six, two, and four cycles of cemiplimab with the palliative intent to treat painful and ulcerated lesions.

**Table 1:** Patients’ demographic characteristics.

Patients	30
Median age, years (range)	81 (36–95)
Sex	
Male	24 (80%)
famale	6 (20%)
ECOG performance status	
0	7 (23.3%)
1	17 (56.7%)
2	6 (20%)
Primary cutaneous squamous cell carcinoma (CSCC) site	
Head or neck	23 (76.7%)
Limbs	5 (16.7%)
Ubiquitous skin lesions	2 (6.7%)
Previous chemotherapy for CSCC	3 (10%)
Previous radiotherapy for CSCC	10 (33.3%)
Previous surgery for CSCC	
0–1 surgery	15 (50%)
2–4 surgeries	7 (23.3%)
More than five surgeries	8 (26.7%)
Histological differentiation of tumor	
Well differentiated	4 (13.3%)
Moderately differentiated	12 (40%)
Poorly differentiated	10 (33.3%)
Unknown	4 (13.3%)
Locally advanced CSCC	25 (83.3%)
Metastatic cutaneous CSCC	5 (16.7%)
Immunosuppressive conditions^a^	5 (16.7%)
Main comorbidities	
Cardiovascular	20 (66.7%)
Metabolic	5 (16.7%)
Respiratory	6 (20%)
Mental disorders	3 (10%)
Frailty score	
Not frail	5 (16.7%)
Frail	25 (83.3%)
Charlson Comorbidity Index	
0	6 (20%)
1	8 (26.7%)
2	7 (23.3%)
3	5 (16.7%)
4	2 (6.7%)
5	1 (3.3%)

Data are n (%), unless otherwise specified.

a Three patients with lymphoproliferative disease and two patients receiving immunosuppressive therapy.

### Clinical Outcomes

**Figure 1:**
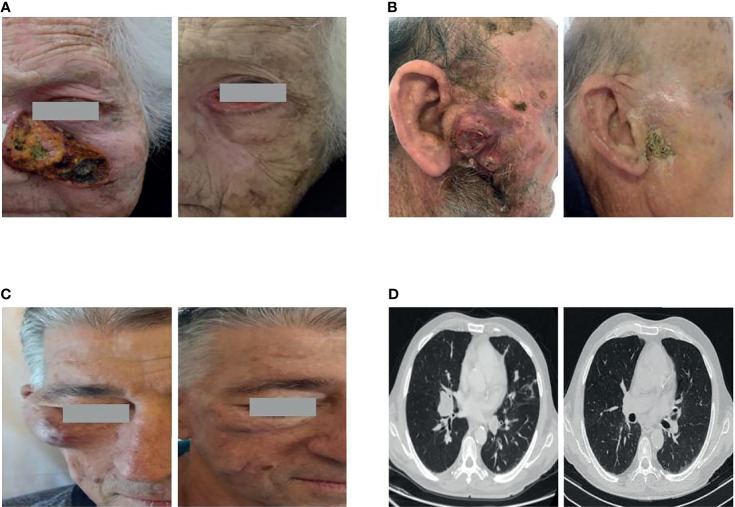
Representative cases of patients obtaining a major response to cemiplimab. **(A)** An 88-year-old female with a large locally advanced cutaneous squamous cell carcinoma (laCSCC) of the left nasal-infraorbital region achieving a complete response. Neither had she received prior radiotherapy nor anticancer systemic therapy. **(B)** An 89-year-old man with a large laCSCC tumor of the right parotid region obtaining a complete response after 6 cycles of cemiplimab and concurrent radiotherapy. **(C, D)** A 67-year-old man with metastatic cutaneous squamous cell carcinoma in immunosuppressive therapy due to a previous kidney transplantation. The patient achieved a near-complete response both at the right zygomatic area and the metastatic lung lesions.

All patients were evaluable for response and safety. An objective response was observed in 23 patients (76.7%, 95% CI: 57.7–90.1), including nine complete responses (30%) and 14 partial responses (46.7%). Moreover, one patient (3.3%) obtained a stable disease for 4 months. Globally, the DCR was 80% (95% CI, 61.4–92.3). Six patients reported progressive disease as best response (20%). The median duration of response was not reached at data cutoff. At present, the longest duration of response is 22 months, and it is still ongoing. Clinical outcomes are summarized in **Table 2**. In a female with bilateral gross preauricular lesions, we observed a pseudoprogression of the right lesion with an initial increase in size followed by a progressively slow decrease to near-complete remission. In **Figure 1**, we reported some representative cases of responsive patients.

**Table 2:** Assessment of tumor response (30 patients).

Response	*N* (%)	95% CI
Complete response, *n* (%)	9 (30)	13.6–46.4
Partial response, *n* (%)	14 (46.7)	28.8–64.5
Stable disease, *n* (%)	1 (3.3)	0.1–17.2
Progressive disease, *n* (%)	6 (20)	7.7–38.6
ORR, *n* (%)	23 (76.7)	57.7–90.1
DCR, *n* (%)	24 (80)	61.4–92.3
Observed duration of response ≥6 months, *n* (%)	18 (60)	
PFS, median (range)	16 (1–23)	
OS, median (range)	18 (1–23)	
Median observed time to response, months (range)	2 (1–5)	

ORR, overall response rate (defined as complete response + partial response); DCR, disease control rate (defined as complete response + partial response + stable disease); PFS, progression-free survival; OS, overall survival.

**Figure 2:**
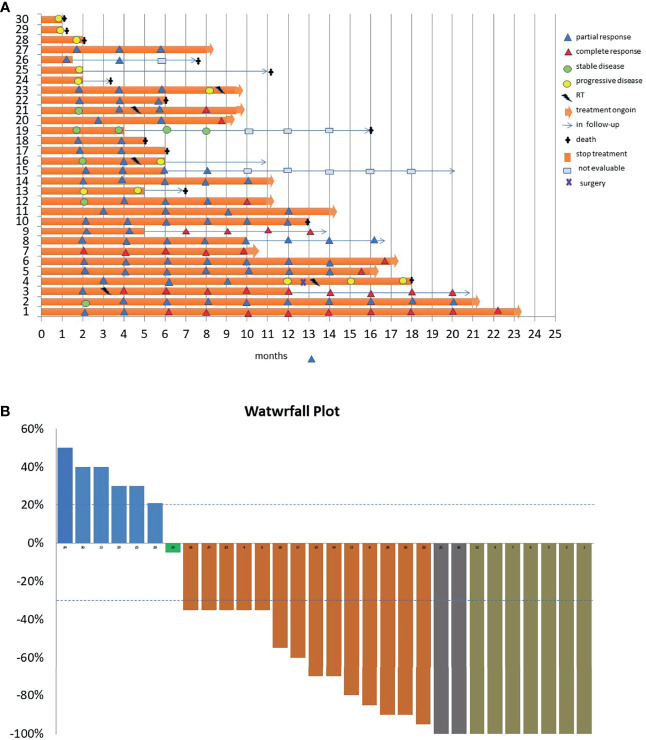
**(A)** Swimming plot showing the time and duration of response (30 patients). Each horizontal line represents one patient. **(B)** Waterfall plot representing the rate of change in target cutaneous squamous cell carcinoma lesions from baseline during the cemiplimab course.

The median time to response was 2 months (range, 1–5). The main characteristics of tumor responses are shown in the swimming plot (**Figure 2A**) and waterfall plot (**Figure 2B**).

**Figure 3:**
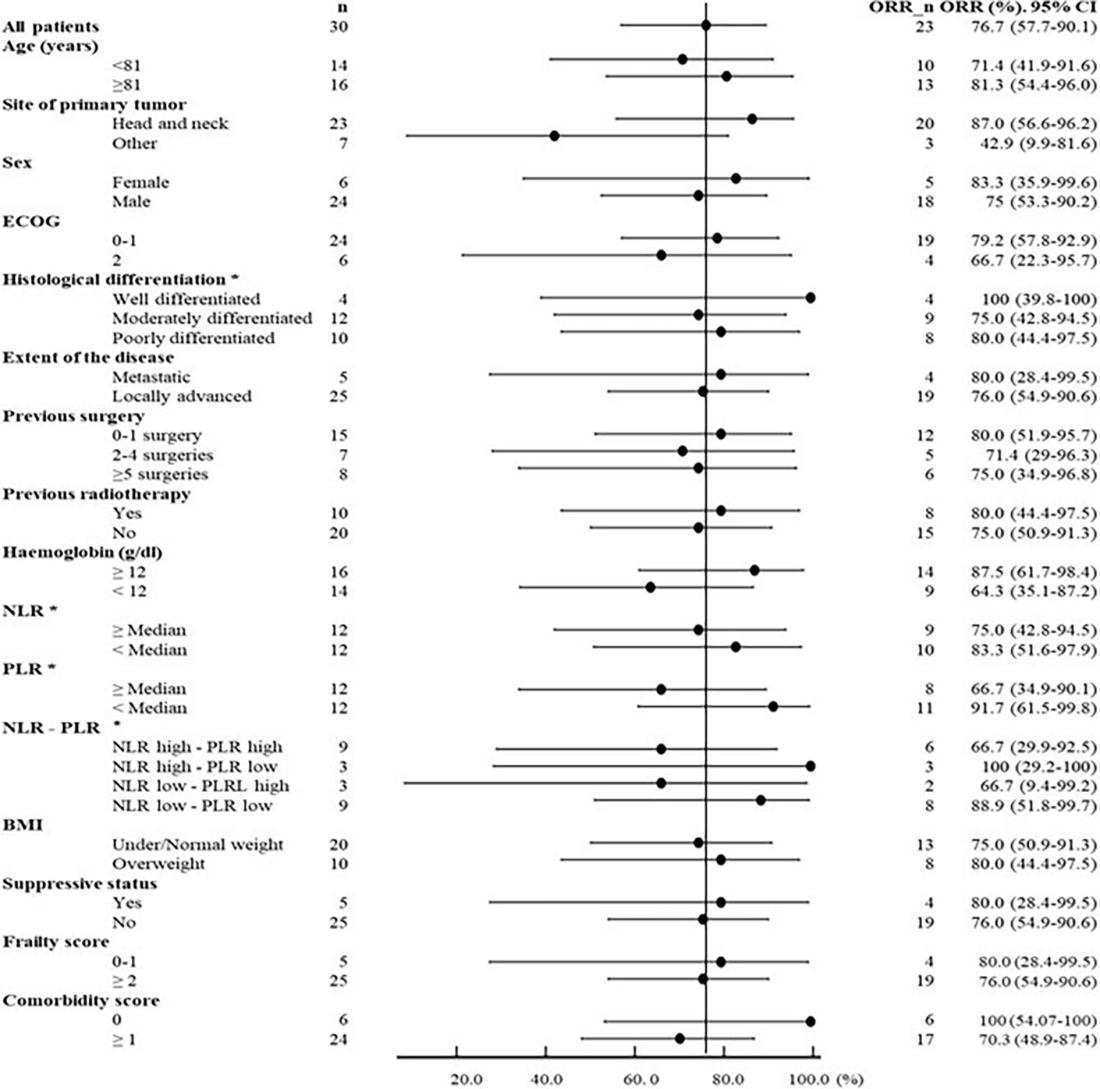
Forest plot of response in selected subgroups of patients according to the main clinical and hematological characteristics. For hematologic parameters, we considered pre-therapy values: only 24 patients were considered eligible (three patients were excluded for concomitant hematological tumors, one for thalassemia major, and two patients for concomitant immunosuppressive therapy).

Regarding correlations between patient/disease features and therapeutic outcomes, we observed a higher ORR in head and neck primaries (87 *vs*. 42.9% of others, *p* = 0.016), in well differentiated histotypes (100%, 95% CI: 39.8–100 *vs*. 75% of moderately and 80% of poorly differentiated), in patients without comorbidity (100%, 95% CI: 54.07–100 *vs*. 70%), and in patients with no or one surgery than in those receiving more than one surgery (80%, 95% CI: 51.9–95.7 *vs*. 71.4 and 75% of two to four surgeries and five or more surgeries, respectively). A modest better response was also reported in patients older than the median age of 81 years (81.3%; 95% CI 54.4–96.0 *vs*. 71.4%, 95% CI: 41.9–91.6), in females (83.3%, 95% CI: 35.9–99.6 *vs*. 75% of male), and in ECOG 0–1 (79.2%, 95% CI: 57.8–92.9 *vs*. 66.7% of ECOG 2) as well as a slight increase in non-frail *vs*. frail (80 *vs*. 76%) and overweight (BMI ≥25 kg/m^2^) *vs*. non-overweight (BMI <25 kg/m^2^) patients (80 *vs*. 75%) (**Figure 3**).

**Figure 4:**
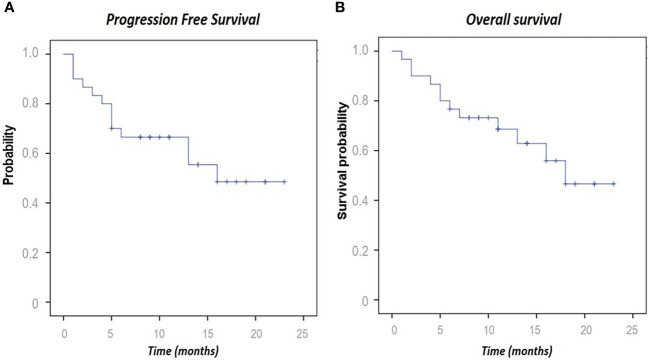
Kaplan–Meier curves for progression-free survival **(A)** and overall survival **(B)**.

Among all patients, the median PFS was 16 months (1–23), and the median OS was 18 (1–23) at the data cutoff date of July 2021. With regard to PFS, 13 events were observed (including nine patients with progressive disease and four deaths). Regarding OS, 13 deaths were reported from enrollment to the data cutoff, providing a 57.6% 10-month OS (**Figure 4**).

### Therapeutic Outcomes in Immunosuppressed Patients

Five patients (16.7%) had an immunosuppressive condition: three stable hematologic malignancies including two chronic lymphocytic leukemia and one idiopathic myelofibrosis previously treated with anti-JAK therapy for about 6 years, while two patients were on immunosuppressive therapy for renal organ transplantation and Crohn’s disease, respectively. Among these five patients, we observed a RECIST response in four patients (80%), including one complete response in the patient with idiopathic myelofibrosis and two partial responses with a tumor shrinkage greater than 80% in the patients with solid organ transplantation and Crohn’s disease. In these patients, the treatment with cemiplimab is ongoing, and the duration of response ranged from 8 to 22 months. Of the two patients with lymphoproliferative disease (B-cell lymphoma and chronic lymphocytic leukemia), one presented a rapid progression and the other progressed after a transient partial response lasting 6 months. Interestingly, no immune-related toxicity was reported in immunosuppressed patients; in particular, no worsening of pre-existing Crohn’s disease as well as no evidence of graft rejection was observed in a kidney transplant patient who, until July 2021, received 20 cycles of cemiplimab achieving a near-complete response (**Figures 1C, D**) and continued immunosuppression with a combination of tacrolimus, sirolimus, and a low dose of steroids.

### Hematological Parameters

The hemoglobin level was analyzed in all patients and correlated with clinical outcomes. The other hematological parameters were collected in only 24 patients (three patients were excluded for concomitant hematological tumors, one for thalassemia major, and two patients for concomitant immunosuppressive therapy).

**Figure 5:**
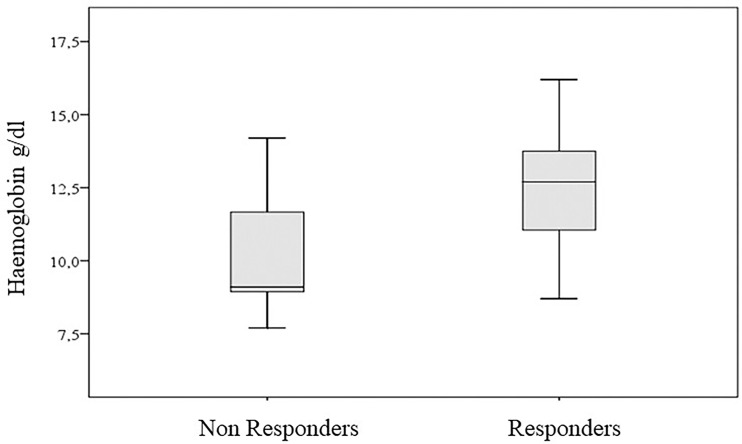
Hemoglobin values according to clinical response.

We found a better response in patients with hemoglobin >12 g/dl (87.5%, 95% CI: 61.7–98.4, *vs*. 64.3% for hemoglobin <12 g/dl). However, when we considered this binary characterization, the association with the response to therapy was not significant (*p*-value of chi-square test equal to 0.134). On the contrary, we found a significant association when we considered the hemoglobin values measured on an interval scale (*p*-value of Wilcoxon–Mann–Whitney test equal to 0.042) (**Figure 5**).

Despite limitations related to the small number of patients, the combined scores of basal NLR and PLR after dichotomization of low PLR correlated to better ORR either in association with high NLR (100% correlation with 95% CI: 29.2–100) and, to a lesser extent, with low NLR (88.9% correlation with 95% CI: 51.8–99.7). Weaker correlations were observed for patients with high basal PLR in association either with low NLR (66.7% association with 95% CI: 9.4–99.2) or with high NLR (66.7% association with 95% CI: 29.9–92.5) (**Figure 3**).

**Figure 6:**
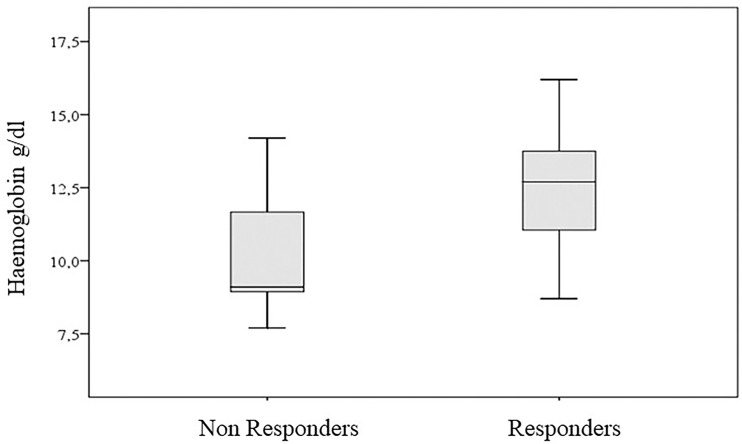
Trends of the main blood parameters according to clinical response. Twenty-four patients were considered eligible (three patients were excluded for concomitant hematological tumors, one for thalassemia major, and two for concomitant immunosuppressive therapy). For non-responders, data from the 6-month sampling are not available.

We also evaluated these blood parameters before therapy and their changes over time. Due to the small number of patients evaluated, the results were not suitable for a statistical test and are reported only in a descriptive manner. The trends of the main parameters considered are summarized in **Figure 6**. Notably, the neutrophils and NLR progressively increased in non-responders compared to responders. Furthermore, the lymphocytes increased slowly during the course of therapy in the responders, while they decreased in the non-responders. The monocytes, already much higher at baseline in non-responders after an initial modest decrease, rapidly increased after 2 months of therapy. Finally, the platelets, already much higher in non-responders at baseline, decreased in both responders and non-responders during cemiplimab therapy. This behavior reflected that of the PLR.

### Safety

Regarding the toxicity profile, the treatment was generally well tolerated by the majority of patients. The most common adverse events included skin toxicity in 10 patients (33.3%), with grade 2 pruritus in six patients, rash in three patients, grade 3 bullous erythema in one patient, and fatigue in seven patients (23.3%). Only three (10%) patients experienced severe grade 3/4 toxicity. A single grade 4 toxicity was observed after the completion of two cycles of treatment in a non-responsive patient with acute respiratory failure due to pneumonitis that required hospitalization and led to death. The treatment-related adverse events are summarized in **Table 3**.

**Table 3:** Treatment-related adverse events (AEs).

Adverse event	Grades 1 and 2	Grade 3	Grade 4
Fatigue	6	1	0
Skin toxicity^a^	9	1	0
Respiratory failure	0	0	1
Interruption with definitive discontinuation due to AEs	0	2	0

Grades are defined as per the Common Terminology Criteria for Adverse Events, version 5.0.

AE, adverse events.

a Pruritus in six patients, skin rash in three patients, and G3 bullous erythema in one patient.

Two patients discontinued the treatment for toxicity despite a response: one with complete response for grade 3 bullous erythema occurring after seven courses of therapy and one with partial response for grade 3–4 asthenia.

Three more patients discontinued treatment due to reasons other than side effects: one in complete response for own personal choice, one in partial response for pre-existing mental conditions compromising her compliance, and the last one in stable disease for rapid worsening of his ECOG (**Figure 2**). All these patients continue to have a response despite the end of the treatment. No additional toxicity on the irradiated lesions was reported in the three patients who underwent concomitant radiotherapy: these patients achieved a response, one of which was complete (**Figure 1**). Beyond eight deaths related to the progression of CSCC, five patients died due to unrelated cancer causes (one death for COVID-19 infection, one for myocardial ischemic attack, one for dementia complications, one for septic complication, and one for cirrhotic decompensation).

## Discussion

Advanced CSCC not amenable to curative surgery and radiotherapy is a severe condition almost always involving elderly and frail patients. In advanced stages, this skin cancer is a disfiguring, painful, and functionally limiting condition that requires a multidisciplinary management to ensure clinically substantial outcomes and preserve the quality of life.

Cemiplimab represented a paradigm shift in these settings, leading to remarkable 44 and 45% response rates associated to durable efficacy in 78 and 115 patients with laCSCC and mCSCC, respectively, according to the results of the phase 2 EMPOWER trials (17, 33).

However, after the approval of this PD-1 blocking agent, several clinical needs are still to be addressed. In particular, these randomized controlled trials underrepresented real-life patients with poor performance status and relevant comorbidities as pathologic or iatrogenic immunosuppressive conditions or organ function deterioration. All these conditions are frequently encountered for CSCC, often involving the elderly population, and delineate a clinical state of frailty characterized by decline across multiple physiological systems that places cancer patients at an increased risk of poor outcomes (18, 19).

In our observational study, we reported a population with a median age of 81 years, which is higher than that reported in controlled clinical trials (71 and 74 years in mCSCC and laCSCC, respectively) and in other real-world experiences (20–24), and a prevalence of frailty of 83%, which is greater than that reported in a metanalysis in generalized (18) and specific oncologic settings (19). Although there is no standard instrument to identify frailty, we set up a frail index based on a simple scoring system that accounts the main tool to assess vulnerability such as age, performance status, and comorbidity. This latest feature was a relevant trait of our population whose median CCI was 2 and accounted five patients with an immunosuppressed status. We observed an unexpected overall response rate of 76% with complete responses of 30%. Despite the poor profile of our patients, these results are better than those reported in controlled trials and initial real-world series showing 31 to 58% overall responses, respectively (14, 20–24). Our better results could likely be due to the prevalence of locally advanced over metastatic stage in our patient population and to the use of cemiplimab as first-line therapy in the majority of patients, which is notoriously associated to better response (34). What is worthy of consideration is that, compared to controlled clinical trials (17, 30) and other real-world series (20–24), our CSCC cohort has been less pretreated even with radiotherapy and surgery. Furthermore, accordingly with other authors, we observed a higher response rate associated with fewer surgical procedures (17) and for CSCC arising in the head and neck area (20–23). This last finding could reflect the influence of a higher degree of sun exposure on the mutational burden notoriously associated with a better response to immuno-checkpoint inhibitors (21, 22). Moreover, we were able to correlate a better response rate with the well-differentiated histological type.

Even with the limitations of the sample size, in the forest plot analysis, we did not find a correlation with response for other clinical features previously described as predictive markers, like male sex (35) and body mass index (36). We likewise found no differences in response between over or under the median age of 81 years. Our data, according with those from similar experiences in melanoma (37), clearly disproves the mistake that age-related impairment of the immune system hampers the effectiveness of PD-1 blockade. This evidence could explain the efficacy of cemiplimab also in the frail subgroup and add data to a poorly investigated issue on which trials are being planned (32). Of note is that we found an equivalent rate of response also in the subgroup of immunosuppressed patients. Other authors also reported responses in CSCC patients who have undergone kidney transplantation or with leukemia (14, 23, 24) as well as in patients treated with immunosuppressive drugs for an autoimmune disease (23, 24). In immunosuppressed patients, the likelihood of a response to PD-1 blockade has been demonstrated also in other cancers (38–40).

Interestingly, simple peripheral blood parameters appeared to be associated both to predicting and assessing response to cemiplimab early. Overall, we found a statistically significant association between pre-treatment hemoglobin levels and response using a threshold of 12 g/dl. As known, hemoglobin levels have a prognostic role in cancers (41) and are predictive for response to various anti-cancer therapies, especially when combined with albumin, lymphocyte, and platelet levels (42). It has been also reported that, regardless of its causes, hemoglobin levels could influence the activation status of T cells against cancer (43, 44). Other authors also reported an association between higher hemoglobin levels and better clinical outcomes both in CSCC and lung cancer patients treated with PD-1 inhibitor (24, 45).

Beyond hemoglobin, we focused on white blood cells whose role as an inflammatory index, influencing response to checkpoint, was established (25). In the baseline evaluation, the combinations of low N/L ratio and low PLT/L ratio appeared as predictors of response, also according with other authors who reported an association between pre-treatment absolute lymphocyte count and response (22). In the longitudinal analysis, we found that the trend of lymphocytes, neutrophils, and monocytes appeared opposite in responder and non-responder patients. If confirmed in a larger population, these data could be relevant to monitor the treatment efficacy early and deserve to be investigated prospectively.

Regarding survival, even if treatments and follow-up are still ongoing in 17 patients, our study showed a trend in PFS and OS comparable to those of previous cemiplimab trials with long duration of response. The proportion of patients who had no disease progression at a median follow-up of 10 months was 57.6%.

The treatment was well tolerated by the majority of patients showing an overlapping toxicity profile with regards of clinical trials. The most common adverse events included skin toxicity and fatigue, with only three patients experiencing severe (grade 3/4) toxicity. Interestingly, three patients who achieved a response and with interrupted treatment due to toxicity or personal choice maintained the response. Moreover, in three patients treated with concomitant radiotherapy, we documented no additional toxicity. This combined therapeutic strategy deserves further investigations due to its interesting biological rational of a synergic action between radiations and immunotherapy with checkpoint inhibitors, as already demonstrated in different types of cancer (46–48).

## Conclusion

In spite of the observational nature of our study and the limited number of patients enrolled, our experience adds evidence on the high antitumor activity of cemiplimab and its safe profile in a broad spectrum of non-selected patients. Moreover, our data offer the possibility of bridging the knowledge gap about cemiplimab performance in a very elderly and frail population.

Nevertheless, some open questions remain to be answered: the weight of the cost/benefit profile of the treatment in peculiar patients such as immunosuppressed patients, patients who have had a transplant, and patients with deteriorated performance status; the identification of biomarkers that could predict efficacy or early assess response to the treatment; the possibility to interrupted treatment at the achievement of a response; the potential combination with local therapy; and the long-term tolerability of the treatment.

## Data Availability Statement

The raw data supporting the conclusions of this article will be made available by the authors, without undue reservation.

## Ethics Statement

The study was previously approved by the ethics committee of the IRCCS Istituto Tumouri Giovanni Paolo II, Bari, Italy (Prot. 590/16 C.E.), and written informed consent was obtained from all the patients enrolled in the study.

## Author Contributions

MG and SS conceptualized the study. IR, LF, DQ, AA, RF, EM, ER, FM, MT, FL, AN, SF, FMa, FF, AS, AAl, ST, and SS participated in data collection. AF and RM contributed to the methodology. SS, AF, DQ, AN, AA, FF, RF, LF, FM, MT, FL, EM, ER, SF, FMa, ST, AS, IR, AAl, RM, and MG participated in analysis, writing, and editing. MG supervised the study. All authors contributed to the article and approved the submitted version.

## Conflict of Interest

The authors declare that the research was conducted in the absence of any commercial or financial relationships that could be construed as a potential conflict of interest.

## Publisher’s Note

All claims expressed in this article are solely those of the authors and do not necessarily represent those of their affiliated organizations, or those of the publisher, the editors and the reviewers. Any product that may be evaluated in this article, or claim that may be made by its manufacturer, is not guaranteed or endorsed by the publisher.
