# Supplementary material for: Cemiplimab in an Elderly Frail Population of Patients With Locally Advanced or Metastatic Cutaneous Squamous Cell Carcinoma: A Single-Center Real-Life Experience From Italy
Source: Front Oncol. 2021 Nov 8;11:686308. doi: 10.3389/fonc.2021.686308 (PMC8606572; doi:10.3389/fonc.2021.686308)
Supplement: Supplementary file 1 [file DataSheet_1.pdf]

Supplementary Table 1. Frailty Assessment. A score of 0-1 defined non-frail patients while a score ≥2 identified frail patients.

Category	Score
Age<br>≤75 years<br>76-80 years<br>>80 years	 0<br>1<br>2
Charlson Comorbidity Index<br>≤1<br>>1	 0<br>1
ECOG PS score<br>0<br>1<br>≥2	 0<br>1<br>2
